# A multistep bioinformatic approach detects putative regulatory elements in gene promoters

**DOI:** 10.1186/1471-2105-6-121

**Published:** 2005-05-18

**Authors:** Stefania Bortoluzzi, Alessandro Coppe, Andrea Bisognin, Cinzia Pizzi, Gian Antonio Danieli

**Affiliations:** 1 Department of Biology, University of Padova – Via Bassi 58/B, 35131, Padova, Italy; 2 Department of Information Engineering, University of Padova – Via Gradenigo 6/B, 35131, Padova, Italy

## Abstract

**Background:**

Searching for approximate patterns in large promoter sequences frequently produces an exceedingly high numbers of results. Our aim was to exploit biological knowledge for definition of a sheltered search space and of appropriate search parameters, in order to develop a method for identification of a tractable number of sequence motifs.

**Results:**

Novel software (COOP) was developed for extraction of sequence motifs, based on clustering of exact or approximate patterns according to the frequency of their overlapping occurrences. Genomic sequences of 1 Kb upstream of 91 genes differentially expressed and/or encoding proteins with relevant function in adult human retina were analyzed. Methodology and results were tested by analysing 1,000 groups of putatively unrelated sequences, randomly selected among 17,156 human gene promoters. When applied to a sample of human promoters, the method identified 279 putative motifs frequently occurring in retina promoters sequences. Most of them are localized in the proximal portion of promoters, less variable in central region than in lateral regions and similar to known regulatory sequences. COOP software and reference manual are freely available upon request to the Authors.

**Conclusion:**

The approach described in this paper seems effective for identifying a tractable number of sequence motifs with putative regulatory role.

## Background

Discovery of regulatory elements in human gene promoters is one of current bioinformatics challenges. Although transcriptional control mechanisms have been investigated in various organisms for at least three decades, it is still almost impossible to predict tissue-specific or developmental-stage-specific expression of a given gene by simply analyzing its promoter sequence [1].

The 5' segment immediately adjacent to the TSS includes the core promoter and the proximal promoter, which usually extends about 200–300 nucleotides. This region is involved in the modulation of transcription. The distal part of a promoter is variable with respect to composition and length, which may encompass from 100 nucleotides to over 2 kb. There is no clear-cut defined 5'-boundary for promoters [2].

Regulatory elements binding the same transcription factor can be found in different promoters as short DNA sequences, differing among them to some extent; they are, in general, from 5 to 25 nucleotides long [3,4], often separated by un-conserved sequences. Control regions are modular in nature and expression of a given gene depends on specific combination of its regulatory elements and sometimes from their order and orientation [5].

Searching by computational methods for promoters and for regulatory elements in DNA sequences spanning several Kb, produces a large number of false-positive results. A possible solution to this problem is to identify a "sheltered environment" in which specificity of pattern discovery might be enhanced. Unknown binding sites for transcription factors might be detected by searching for common elements in upstream regulatory regions of genes with common biological function and/or expression. In fact, genes with similar expression are frequently co-regulated and genes with related function are often similarly expressed [6].

In this study, we attempted to detect putative regulatory elements in promoters of genes expressed in an adult human tissue (retina), by a multi-step approach involving computational analysis of large-scale expression data, selection of a subset of putatively co-expressed genes, retrieval of the upstream portion of their complete genomic sequence and application of pattern discovery on promoter regions.

## Results

### Analysis of known regulatory sequence elements binding transcription factors

Before applying COOP software on a selected group of promoters, we attempted to exploit information on known regulatory sequences available in TRANSFAC [10], to establish some "rules" which could facilitate the discovery of novel regulatory elements. In particular, TRANSFAC matrix data were analysed in order to describe number, percent and localization of fixed and variable positions in consensus sequences.

We first considered 385 matrices including information on mammalian regulatory elements. Average length of consensus sequences was 13.0 and mode 12; motifs of even length were more represented (even lengths two times more represented than odd lengths among consensus sequences of length ranging from 8 to 17). Less than 5% of the motifs showed only invariant positions (average and mode of length of completely invariant motifs were 10.3 and 9, respectively). About 33% of motifs showed more than 75% fixed positions (average length 11.5, mode 10), whereas about 73% showed more than 50% fixed positions (average length 12.3, mode 8). In general, the shortest the motif, the less variable appeared its consensus sequence.

By separately considering three regions of consensus sequences (left, center and right), we observed that lateral positions are variable in 37% of sequences, whereas central positions are variable only in 20% of them.

Most regulatory elements included in TRANSFAC seem to be symmetrical, being equally variable in their left and right sides.

We obtained very similar conclusions from the analysis of the group of 610 eukaryotic matrices. Results of this analysis suggested that pattern discovery on mammalian promoter sequences might focus on patterns 10, 12 or 14 nucleotides long, showing from 0% to 25% variable positions, and possibly, less variable in the central region.

### COOP : Clustering Overlapping Occurrences of approximate Patterns

**Figure 1:**
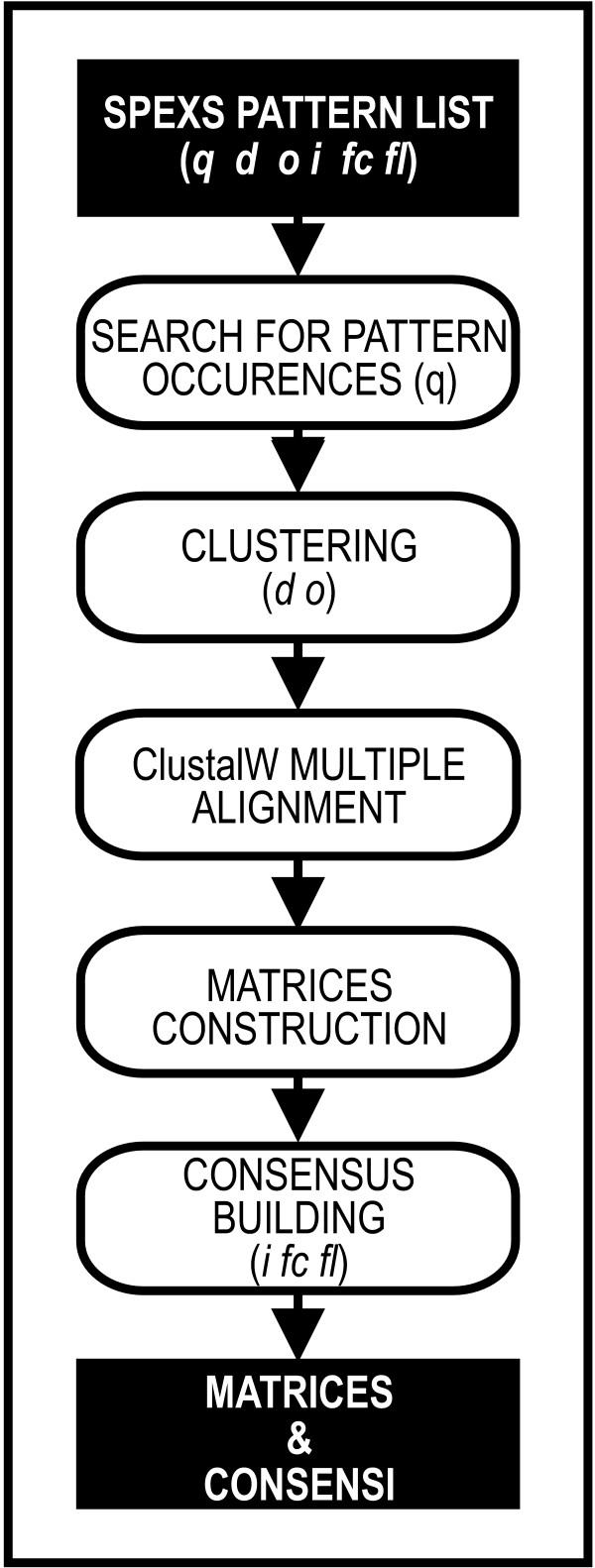
**Flow-chart of COOP program**. Input, output and main steps are shown.

Since sequence signals with biological significance are frequently subtle, stringency of pattern discovery analyses in biological sequences cannot be set too high. This implies that results are often too numerous. A novel tool for Clustering Overlapping Occurrences of approximate Patterns (COOP) was implemented in Python (Figure 1). This software allows identification of tractable numbers of possibly interesting motifs, starting from large numbers of exact or approximate patterns.

### Selection of genes and retrieval of putative promoter regions

Among 1,814 genes expressed in retina, statistical analysis of differential expression, by Audic and Claverie test [11], picked out 80 genes significantly more expressed in retina than in all other tissues. We selected as well a group of 59 known genes whose mutation is known to cause retinal diseases, recorded in OMIM and/or in RetNet databases, and/or encoding proteins for which a specific function in retina has been described. In total, 129 were selected. For each of these genes, the Reference Sequence or the longest sequence of the mRNA with complete CDS was compared by BLAT [12] to human genome sequence, for annotation of the intron/exon structure and for prediction of the most probable TSS (Transcription Start Site). We predicted with good confidence TSS of 90 genes (45 overexpressed UniGene clusters, 28 retinal disease genes, 7 both overexpressed and retinal disease-genes and 10 genes encoding proteins with a retinal function)[13]. Sequences from 90 selected genes, each corresponding to 1 Kb upstream the predicted TSS, were retrieved for further analyses. For one gene, USH3A (LL 7401) two alternative promoters (USH3A-A and USH3A-B) controlling transcription of messenger RNA encoding retinal products were found. Therefore, 91 gene promoters pertaining to 90 different retinal genes were considered for the study.

### Search for approximate patterns

#### Retinal datasets

We considered datasets including sequences corresponding to the 500 bp upstream the predicted TSS of the selected genes (unmasked sequences, 500U91; partially masked, 500PM91; masked, 500M91) and the group of 91 fully masked sequences corresponding to 1 Kb upstream the predicted TSS (1000M91 dataset). Fully masking of sequences in datasets 500M91 and 1000M91 produced, on average, 435 and 788 unmasked nucleotides, respectively.

**Figure 2:**
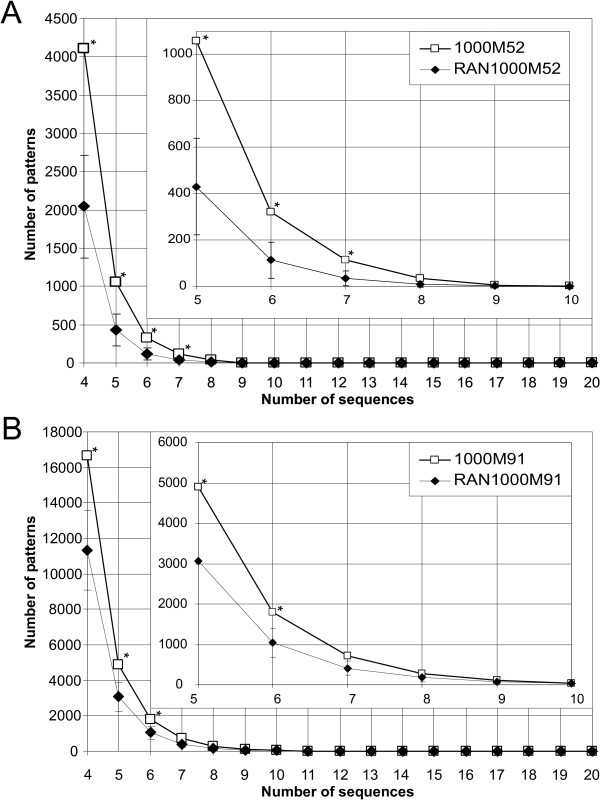
**Comparison of patterns discovery results in retinal gene promoter sequences and in 1,000 negative control datasets**. Plots of number of patterns (12 bp long, with at most two variable positions) *vs *number of sequences in which they were found, in retinal gene promoter sequences (open squares) and in 1,000 negative control datasets (filled diamonds). For negative control datasets, the average value of 1,000 sets of sequences is given, with a two standard deviations interval. Statistically significant differences (0.05 threshold) are marked by stars. (A) Comparison between the 1000M52 dataset (52 promoter sequences of genes overexpressed in the retina) and the RAN1000M52i dataset (1,000 groups of 52 randomly chosen human promoters); (B) Comparison between the 1000M91 dataset (91 retinal gene promoter sequences) and the RAN1000M91i (1,000 groups of 91 randomly chosen human promoters).

In each group of sequences, approximate patterns of length ranging from 10 to 14 nucleotides, with at most two variable positions (10-2, 12-2, 14-2 patterns), were searched by SPEXS (Tables 3 and 4). For each dataset, patterns were ranked in different classes, according to the number of sequences in which they were represented (Tables 3 and 4, Figure 2).

In the 500 bp datasets the number of sequences in which most represented patterns were found as relatively low, reaching a maximum of 44 (48%) for 10-2 patterns in unmasked sequences (500U91) and only 5 (5.5%) for 14-2 patterns in masked sequences (500M91). When considering sequences of length 1,000, the number of sequences with occurrences of most represented patterns slightly increased. For instance, most represented 10-2 patterns were found in 32 sequences in 500M91 and in 40 sequences in 1000M91.

#### Negative control datasets

Random groups of human promoter sequences were established as negative control. One thousand groups of 91 promoter sequences randomly selected among 17,156 (1 Kb long) human gene promoters were generated (RAN1000M91i, with *i *ranging from 1 to 1,000). Each of these 1,000 groups included the same number of sequences of the 1000M91 set of retinal gene promoters and sequences fully masked and of the same length of retinal gene promoters. Moreover, TSS predictions were done by Promoser [14,15] according to identical criteria adopted for TSS prediction of retina genes.

Results of pattern discovery in the 91 retina gene promoters group (1000M91) and in the dataset of 1,000 groups of 91 human gene promoters randomly selected among 17,156 (RAN1000M91i) are shown in Tables 3 and 4 and in Figure 2. The number of patterns with different *quorum *in the retinal datasets and in the negative control groups of promoter sequences are reported. In the last two rows of Table 4 the comparison of pattern discovery results in the 1000M52 retinal gene promoters group and in the dataset of 1,000 groups of 52 sequences (RAN1000M52i) randomly selected among 17,156 human gene promoters is shown.

The number of patterns found in at least 10 and in at least 20 out of 91 retinal promoter sequences is higher than the average of number of patterns found in at least 10 and in at least 20 out of 91 randomly selected promoter sequences, calculated over 1,000 different samples. Statistical significance (P-value) of number of patterns found in each number of sequences of the retina datasets was calculated as the proportion of negative control random datasets in which the number of patterns found was higher or equal to those found in retinal promoters. When this number was below 0.05, the difference was considered statistically significant. Over 1,000 random samples, 351.3 10-2 patterns were found in average in at least 20 promoter sequences, while 719 patterns were found in at least 20 retinal promoters (P-value = 0.017). Similarly, 714 12-2 patterns were found in 7 or more retinal promoter sequences, whereas 410.2 were found in average in random samples (P-value = 0.060). Statistical significance of this observed difference resulted just above 0.05. This effect could be due to possible heterogeneity of the class of 91 gene promoters. When considering the group of 52 promoters corresponding to genes significantly more expressed in retina than in all other considered tissues (1000M52) and the corresponding negative control dataset (RAN1000M52i), the difference between retinal and random datasets is more evident. For instance, 114 12-2 patterns were found in 7 or more retinal promoter sequences, whereas 33.7 in average were found in 1,000 random samples (P-value = 0.027).

In Figure 2A, the cumulative number of patterns is plotted against the number of sequences in which they were found, among sequences of dataset 1000M52 and RAN1000M52i. In Figure 2B the comparison between the 1000M91 and the RAN1000M91i is shown. For negative control (random groups of promoters), the average value of 1,000 sets of sequences is given, with an interval of two standard deviations centred to the mean (e.g. 4,895 patterns were found in at least five sequences out of 91 in the retina dataset, whereas the average number of patterns found in at least five sequences out of 91 was 3,057 in the negative control dataset). It should be noticed that in both comparisons the number of patterns found in the retinal dataset is always considerably higher than average in the negative control dataset.

### Putative novel regulatory elements in retina gene promoters identified by COOP

We considered the group of 716, 12-2 patterns detected by SPEXS in at least 7 out of 91 promoter sequences of genes expressed in human retina (dataset 1000M91). We clustered the 6,611 occurrences of 716 selected patterns by using different sets of parameters, in order to identify combinations maximizing the biological meaning of resulting motifs. Distance parameters ranging from 2 to 5 nucleotides were used, each associated with *o *values of 0.6 or 0.7. The number of clusters decreases when *d *increases and increases with *o*. For instance, when *o *is set to 0.6, the increase of *d *from 2 to 5 changes the total number of clusters from 211 to 183, changing the number of clusters containing only the occurrences of one SPEXS pattern ("single clusters") from 136 to 116, with maximum number of patterns per cluster increasing from 119 to 180. We kept *o *high, in order to cluster patterns according to their "very frequent" overlapping occurrences. The change of *o *from 0.6 to 0.7 changes the number of clusters obtained with d = 3 from 195 to 279, while the number of "single clusters" changes from 123 to 183. The number of clusters containing the occurrences of different SPEXS patterns is quite stable in respect to variation of *o *(72 and 97, respectively), whereas the number of "single clusters" increases considerably with *o*. In all cases, the number of extracted motifs, ranging in these experiments from 169 (*d *= 5, *o *= 0.6) to 300 (*d *= 2, *o *= 0.7), is considerably lower than the number of patterns considered.

Starting from 6,611 occurrences of 716 selected patterns found in at least 7 sequences with *d *set to 3 and *o *to 0.7 COOP produced 279 clusters with an average number of occurrences per cluster of 13.9 (median 9 and mode 7, maximum 150). Clusters are associated to 279 sequence alignments, 89 of which (31.9%) are longer than 12 nucleotides, and to position-specific scoring matrices. In average, 13.9 occurrences per motif were observed. These 279 motifs occurred in 87 sequences out of 91. The mean number of motifs per sequence is 26.7, whereas the mean number of motif occurrences per sequence is 42.6. Subgroups of these motifs could be similar or overlapping.

Motifs occurrences were ranked into four classes according to their position in promoter sequences (bp distance from the predicted TSS). The observed distribution significantly deviated from expectation under assumption of randomness (1,558 motif occurrences from -1 to -250; 1,002 in -251/-500; 626 in -501/-750 and 690 in -751/-1000; chi squared test: P = 3.03*10–121). There is a positive correlation between density of motif occurrences and proximity to TSS. In fact, over 40% of total motifs occurrences are concentrated in 250 nucleotides close to the predicted TSS and the two thirds of the total number of occurrences fall within 500 nucleotides adjacent to the first exon. Regions of promoters sequences corresponding to the 500 bp proximal to TSS were in average less masked than the upstream regions. When normalizing the number of motifs occurrences to the percentage of unmasked nucleotides in the different regions, a strong difference remains, with significant deviation from a random distribution. P value of chi squared test for the comparison among two groups (from -1 to -500 and from -501 to -1000) is 3.3*10–48.

From each sequence alignment pertaining to a cluster, a consensus sequence representing the motif was built. Different sets of parameters were used to this purpose. The choice of different stringency of parameters for building consensus sequences influences the length of obtained motifs and the fraction of variable positions included in them. Threshold *i *ranged from 0.1 to 0.5 and *f_l_* and *f_c_* ranged from 0.6 and 0.8 (data not shown). If *f_l_* and *f_c_*are sufficiently stringent, a low value for *i* could be used, in order to maximize information extracted. When a *i *= 0.1 is applied for construction of consensus sequences, the average length of motifs remains almost unchanged (12.4), with 89 motifs of length over 12. Part of the information about each motif is lost when a matrix is built from the alignment of pattern occurrences and when frequency data are converted into a consensus sequence. We used moderately stringent thresholds for the minimum frequency of a single nucleotide determining "fixed" positions. In particular, *f_l_* was set to 0.5, whereas *f_c_*, referring to the core regions of consensus sequences, was set to 0.7. Out of 279 motifs, only 62 "most informative" motifs were selected, which showed a completely conserved consensus or a consensus showing variable positions only in the side regions (Table 5). The average length of this group of sequence motifs was 13.0, with about 55% of them longer than 12 nucleotides. Two pairs of motifs were represented by the same consensus sequence. The resulting group of 60 motif consensus sequences, representing putative functional elements in retinal promoters, were compared with TRANSFAC data, by TESS program [16]. In particular, we used the "Filtered String-based Search Query" tool [17] for comparison only with known regulatory sites of mammals, with no mismatches allowed and by using the entire length of known sites instead of their core positions. Out of 60 motifs, 53 (88%) exactly matched at least one sequence known to bind a mammalian transcription factor. Sequences corresponding to common or general transcription factors (Sp1, Sp3, MAZ, GCF, CUP or Yi) were matched by 47 out of 60 consensus sequences (78%), 22 of which matches also additional factors (AP-1, AP-2, WT1, Krox-20, GR, PPUR or ER). In total, 26 consensus sequences resulted similar to sequences recognized by AP-1, AP-2, WT1, Krox-20, GR, PPUR or ER.

### Analysis of positive control datasets

To the purpose of both analysing COOP efficiency with selected conditions and of indirectly comparing the performances of the method with those of different software, we analysed by COOP a group of 26 human positive control datasets prepared by Tompa and colleagues for the assessment of computational tools for the discovery of transcription factor binding sites [18,19]. This benchmark included 26 groups of promoters, for which it is known which regulatory signals should be detected.

The number and the length of promoter sequences and of known signals per dataset are reported in Table 6, along with the adopted quorum [see Additional file 1]. The number of signals per group of promoters which are shorter than the length of approximated patterns taken as input by COOP (12 nucleotides) and which were, in principle, very difficult to find by adopted settings, is also reported. For each experiment, among different motifs predicted we selected only the one corresponding to the motif represented in the highest number of sequences. For each dataset the nucleotide-level and site-level [18] overlap between pattern occurrences belonging to the cluster (i.e. the selected motif) and known signals, evaluated by different measures (see Methods), are included in Table 6 [see Additional file 1].

**Figure 3:**
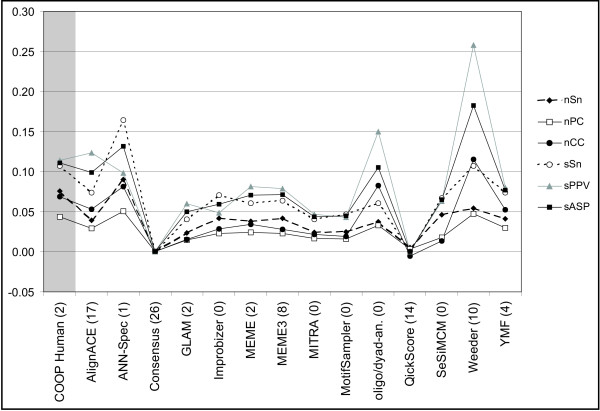
**Statistics comparing the accuracy of COOP and of 14 different motif discovery tools on 26 human positive control datasets**. Combined measures of correctness over all 26 human datasets, as defined in Methods. The number of datasets (out of 26) for which no motif was predicted by each tool is reported in brackets, following the name of the tool.

Moreover, the "combined" statistics summarizing COOP performance over the collection of human datasets, was compared with the same statistics calculated for the 14 different programs tested by Tompa and colleagues on the same datasets [18]. Results are presented in Figure 3 and in Table 7 [see Additional file 2].

The comparison showed that the performances of COOP on the "very difficult" human dataset are in line with those of the top rated tools. In comparison with other software, COOP resulted to be in the best 20% of evaluated tools according to four measures (nSn, nPC, sSn, sASP) and in the best one third according to six measures (nSn, nPC, nCC, sSn, sPPV, sASP).

## Discussion

Pattern discovery in sequences of putative and sometimes incomplete promoters is a considerably complex problem [20-25]. It may be reasonably assumed that some regulatory regions of a group of co-regulated genes share similar sequence elements. In yeast, pairs of genes showing over 0.84 Pearson correlation between their expression profiles, have over 50% probability of sharing at least one common transcription factor binder [26].

Since patterns with biological significance could be subtle [27], a main difficulty in pattern discovery approaches is a priori establishing a "quorum" and defining search parameters (e.g. pattern length, number of allowed wildcards or distance of occurrences from the model). By increasing the distance and/or decreasing the quorum, the number of false positives becomes excessively large. A possible solution to the problem of output explosion is to use biological knowledge both before and after application of automated pattern discovery.

We analysed TRANSFAC data to obtain information about frequent properties of regulatory elements. It should be noticed that although not all TRANSFAC matrices are based on high-quality data and on large samples of sequences, this database represents the largest existing collection of known regulatory elements in different organisms.

In this study we developed a novel tool, called COOP, for analysing promoter sequences of putatively co-regulated genes, aiming at extraction of sequence motifs with possible regulatory function. The motif extraction method is based on Clustering of Overlapping Occurrences of approximate Patterns, which allows identification of tractable numbers of possibly interesting motifs, starting from large numbers of exact or approximate patterns.

Our method is somehow related to two approaches proposed by van Helden [28] and by Caselle [29], although these studies considered yeast promoter sequences and dealt with exact patterns. The originality of our approach, mainly resides principally in adopting a new similarity measure between patterns, based on the frequency of pattern co-occurrences, and in designing a flexible procedure, with seven parameters which could be varied in order to modulate stringency of different analysis steps.

Motifs reconstruction was designed to maximize information included in each extracted sequence motif avoiding generation of spurious elements, given that clustering parameters (*d *and *o*) are appropriately set. Each obtained motif is represented by a consensus sequence, derived from the alignment of strings grouped in a specific cluster by adjustable criteria. In particular, the *i *threshold affects the length of the consensus sequence, whereas the *l*, f_c _and f_l _determine the number and the distribution of variable positions in the consensus sequence.

In order to evaluate the performance of the method, we analysed positive control datasets, such as all the human benchmark groups of promoter sequences, containing known signals at known positions, proposed by Tompa for a systematic assessment of motif discovery tools. COOP analyses were carried out with the same settings used for analysing retina gene promoter. The quorum was established by using an unique criterion for different datasets, based on the total number of sequences in the sample. In the first analysis phase, approximated patterns of 12 nucleotides, with at most 2 variable positions, were searched. Benchmark datasets were 26 groups of different number of promoters sequences. Each set of sequences contained a group of known signals. Lengths of signals ranged from 4 nucleotides to 71 nucleotides. Thus, several datasets contained a number of very short signals, which were very hard to find by a motif discovery approach designed to find motifs of length equal or higher than 12 nucleotides. We predicted a motif for all but two human datasets, which included only two sequences. COOP performed comparably well than the tools which were top rated in the Tompa assessment. It should be noticed that some of the other tools gave no predictions for a number of datasets, thus being advantaged from the averaging nPPV, sPPV and nCC scores when calculating combined statistics over all the human datasets [18].

The method we developed was applied, in a case study, to a collection of human promoter sequences pertaining to a group of 91 putatively co-regulated genes expressed in the retina.

One Kb long promoter sequences were identified by predicting the most probable TSS according to the consensus of information about cDNA and ESTs alignments with the genome sequence. Even neglecting the possible presence of alternative promoters, definition of exact(s) TSS it is a still open problem, because of low sensitivity of promoter prediction programs and of incomplete cDNA coverage of 5' exons. However, cDNA coverage of the majority of genes selected for this study is almost complete, since genes considered are well known and/or highly expressed. The adequacy of our method for selecting gene promoter sequences is supported by results obtained by Trinklein and colleagues [30]: over 90% 152 human 600 bp promoter sequences, randomly selected among 10,276 TSS predictions (based only on alignment with full-length cDNA clones from Mammalian Gene Collection) resulted active in at least two cell lines. Although the size of 1 Kb upstream the TSS might be insufficient to cover all possible regulatory regions for all genes, it could reasonably include both core and proximal promoters and at least part of the distal promoter.

A very high number of approximate patterns of length from 10 to 14 with at most 20% variable positions was found in retina promoter sequences. This is partially due to the fact that they include similar sequences or sequences shifted only by few positions. By using COOP for clustering approximate patterns, on the basis of their frequent overlapping occurrences, we identified a number of interesting sequence motifs, often longer than the original patterns.

In order to test the significance of the group of sequence patterns frequently found in retinal promoters and used as input for COOP, we generated and studied different negative control datasets corresponding to a thousand groups of sequences, randomly selected among a very large group of human promoters. The average pairwise level of pattern sharing in these groups of promoters was expected to reflect the general level of pattern sharing between human gene promoters. We observed that the selected group of retinal promoters (pertaining to a sample of genes putatively co-expressed, co-regulated and/or with similar function) is enriched in common patterns as compared to random groups.

Sequence motifs produced by COOP resulted more frequently in the regions close to the TSS. Moreover, the group motifs consensus sequences selected according to very low variability in their central region was compared with sequences which are known to bind mammalian transcription factors under stringent criteria. Signals similar to those for general and widespread transcription factors, such as Sp1 or MAZ, are the most represented. Moreover, a number of selected motifs resulted to be similar to signals recognised by transcription factors expressed in tissues of ectodermal origin and relevant to development and function of retina (AP-1, AP-2, WT1, Krox-20, GR, PPUR or ER). For instance, AP-1 elements were found in a number of retinal gene promoters including cGMP-phosphodiesterase beta subunit [31] and hydroxyindole-O-methyltransferase [32] whereas the WT1 zinc finger factor is essential for normal development of retina and specifically involved in regulation of retinal genes [33].

## Conclusion

We developed a method to detect sequence motifs corresponding to putative regulatory elements in gene promoters, starting from lists of approximate patterns with occurrences in promoter sequences. This method could be profitably applied to different datasets, including promoter sequences of different groups of genes in humans or in other Eukaryotes, for which co-regulation could be demonstrated or inferred. The method could be used to investigate on different kinds of regulatory sequences, such as intronic enhancers, or other sequence motifs with non-regulatory function.

## Availability and requirements

COOP can be downloaded free-of-charge from the web page . COOP was developed in Python. The software works under Linux and requires Python 2.3 or higher, BioPhyton 1.40b and ClustalW. COOP is provided 'as is' with no guarantee or warranty of any kind and it is freely available for all.

## Methods

### COOP : Clustering Overlapping Occurrences of approximate Patterns

COOP takes as input a FASTA file of nucleotide sequences and a list of patterns with their number of occurrences in sequences or in their reverse complement. We used the SPEXS program [7] for producing the list of approximate patterns frequently represented in selected groups of promoter sequences. SPEXS code is freely available and it provides a number of advantages in terms of execution time and flexibility of parameters determining search conditions and output appearance.

Seven COOP parameters can be varied in order to select stringency at different stages of the analysis (Table 1).

In the first step, patterns represented in more than *q *promoters are searched by COOP in promoter sequences. In particular, both direct and reverse complement sequences of each pattern are compared against promoter sequences in order to collect pattern occurrences. Then, pattern occurrences (strings) are clustered according to a similarity measure based on frequency of their co-occurrences and by a joining algorithm derived from the so called "quick-find" algorithm [8]. In order to be included in the same cluster, two different strings must occur in promoter sequences much more frequently together than separately. Given the physical distance between pattern occurrences, measured as nucleotide distance between the 5'-ends of two corresponding sequences, the threshold *d *defines the maximum value for the distance between two pattern occurrences to be considered overlapping. Threshold *o *indicates the minimum ratio between observed overlapping occurrences of two strings and their average number of occurrences, allowing their inclusion in a unique cluster. The total number of clusters obtained in this way is influenced by the number of pattern occurrences to be clustered, depending on the *q *parameter, and depends as well on values selected for the minimum distance between patterns and for threshold *o*.

Once clusters are obtained, all sequence elements corresponding to pattern occurrences belonging to each given cluster are multi-aligned by ClustalW [9], with Gap Opening Penalty set to 100. Each alignment is then analysed in order to build up a matrix describing nucleotide counts in alignment positions. The cell *x*_*A*1 _of the matrix contains the number of times the A nucleotide has been observed in the first alignment position. Later, consensus sequences are built from matrices (Table 2). The maximal number of adjacent matrix positions fulfilling the established threshold for *i *(minimum ratio between number of nucleotides per alignment position and total number of lines in the alignment) is further analysed to determine the *m *nucleotides long motif consensus sequence.

Once the extension of lateral regions (*l*, ranging from 0 to m/2) is fixed, motif positions are considered invariable if the frequency of a single nucleotide exceed selected thresholds (*f_l_* for lateral regions and *f_c_* for the core region of the motif). Once the length of motif and of lateral regions is known, the extension of the core region is fixed.

Moreover, an additional procedure is available, which uses IUB/IUPAC nucleic acid single letter, double-degenerate codes (M = [A|C], K = [G|T], S = [G|C], W = [A|T], R = [G|A], Y = [T|C]) and four-degenerate code (N = [A|G|;C|T]) and follows IUPAC rules for string consensus reconstruction: i. A single nucleotide is shown if its frequency is greater than 50% and at least twice as high as the second most frequent nucleotide; ii. A double-degenerate code indicates that the corresponding two nucleotides occur in more than 75% of the underlying sequences (but the criteria for a single nucleotide assignment are not met); iii. All other frequency distributions are represented by the letter "N".

The output of the program is a collection of sequence clusters, each one representing a sequence motifs. Each cluster is associated to an alignment, to a matrix describing nucleotide counts in alignment positions and, ultimately, to a consensus sequence. Moreover, information about promoter sequences and nucleotide positions in which each cluster string occurs is given.

### Selection of genes and of putative promoter regions

#### Analysis of genomic expression data

For the study, genes significantly more expressed in retina than in all other tissues were identified by analysis of genomic expression profiles of several human tissues. Genomic expression profiles were reconstructed *in silico *by using 41 unbiased (un-subtracted and/or un-normalized) UniGene cDNA libraries pertaining to 11 adult human normal tissues (retina, bone, hyppocampus, liver, lung, marrow, melanocyte, muscle, pancreas, prostate and testis) for which at least 6,000 ESTs per tissue were available [34]. The whole dataset included 270,871 ESTs, corresponding to 27,924 UniGene "clusters". The expression profiles were merged in an expression data matrix, which was then analysed by the Audic and Claverie test of differential expression in order to identify genes significantly more expressed in retina than in all other tissues considered. Significance threshold was set to a = 0.05.

#### Disease genes and genes encoding proteins with a specific function in the retina

By searching in OMIM [35], Retinal Information Network [36], LocusLink [37] and GeneCards [38,39] we selected a group of known genes whose mutation causes retinal diseases and genes encoding proteins which play specific functions in retina.

#### Retrieval of putative promoter regions

Reference Sequences of selected genes (RefSeq) [37] were extracted from corresponding LocusLink entries. When RefSeq for a gene was unavailable, the longest mRNA sequence with complete CDS was used. Sequences were then searched by BLAT [40] against release 15 of human genome sequence, for prediction of Transcription Start Site (TSS), obtained by analysis of mRNA/genomic DNA alignment, 5' ESTs placement and Acembly gene boundaries [41] annotation. Genomic sequences of 1 Kb upstream the predicted TSS were retrieved. These sequences were masked by RepeatMasker [42], in order to remove repetitive DNA.

### Negative controls

Negative control groups of promoter sequences were established as 1,000 sets of promoter sequences, sampled at random among 17,156 human gene promoters.

Reference sequences of 27,427 human mRNA were obtained from GenBank. A group of 20,315 reference sequences was obtained, after exclusion of all sequences referring to unknown genes (chromosome open reading frames, hypothetical or predicted proteins) or to genes including in their sequence record words referring to vision, eye or retina.

Retrieval of promoter sequences corresponding to 1 Kb upstream the predicted TSS of genes was done by PromoSer [14,15]. TSS prediction options were set in order to retrieve for each gene 1 Kb upstream the most 5' TSS, with the same criteria used for retrieval of retinal gene promoters. In addition, exclusion options of PromoSer were set to extract at most one promoter per gene and to avoid retrieval of overlapping sequences. By this way, 17,156 promoters were localized and retrieved.

A Python script was developed for iterative random extraction of groups of m sequences from a list of N sequences, with N > m, without repetition.

### Analysis of known regulatory sequence elements binding transcription factors

We used the TRANSFAC version available through BIOBASE. TRANSFAC [43] is a database of eukaryotic cis-acting regulatory DNA elements and trans-acting factors containing information on transcription factors, regulated genes, regulatory sites and nucleotide distribution matrices for binding sites of transcription factors. By using Perl scripts developed to the purpose, flat files pertaining to matrices data were parsed in order to extract information about consensus sequences length and about number, percent and localization of fixed and variable positions.

### Analysis of positive control datasets

We analysed by COOP groups of promoters for which it is known which regulatory signals should be detected. Analyses were done of the complete set of groups of human sequences included in public benchmarks prepared by Tompa and colleagues [18], including three different types of sequences: 9 groups of real genomic promoter sequences containing known transcription factors binding sites, 9 groups of randomly chosen human genomic promoter sequences in which the binding sites were planted and 8 groups of sequences randomly generated according to a Markov chain of order 3 (that was constructed from human promoter sequences) in which the binding sites were planted.

We analysed such datasets by using the same methodology applied for the identification of putative novel regulatory elements in retinal gene promoters, including searching for 12-2 patterns by SPEXS and motifs reconstruction by using COOP (clustering parameters: *d *= 3 and *o *= 0.7). The quorum *(q*) was set to the highest integer equal or less than one third of the total number of sequences in the dataset. When with the selected quorum no results were obtained, *q *was set to the highest integer giving results. When analysing datasets composed of from 3 to 6 sequences, *q *was set to *2*. We made no motif predictions for the two datasets of two sequences each.

For each group of promoters, among different clusters obtained, we selected only the one corresponding to the motif represented in the highest number of sequences. For each dataset out of the 26 considered, we checked the overlap between pattern occurrences belonging to the cluster (i.e. the motif) and known signals. The efficiency of COOP was evaluated according to different measures, defined as follows. At nucleotide-level nTP (number of nucleotide positions in both known sites and predicted sites), nFN (number of nucleotide positions in known sites but not in predicted sites), nFP (number of nucleotide positions not in known sites but in predicted sites) and nTN (number of nucleotide positions in neither known sites nor predicted sites). A predicted site overlaps a known site if they overlap by at least one-quarter the length of the known site. Thus, at site-level we calculated: sTP (number of known sites overlapped by predicted sites), sFN (number of known sites not overlapped by predicted sites) and sFP (number of predicted sites not overlapped by known sites). We then calculated the following measures of accuracy. At either the nucleotide (x = n) or site (x = s) level: Sensitivity, xSn = xTP/(xTP + xFN); Positive Predictive Value, xPPV = xTP/(xTP + xFP). At the nucleotide-level: Specificity, nSP = nTN/(nTN + nFP); Performance Coefficient, nPC = nTP/(nTP + nFN+ nFP); Correlation Coefficient, ; Average Site Performance, sASP = (sSn + sPPV)/2 [18].

In addition, the statistics (nSn, nPPV, nSp, nPC, nCC, sSn, sPPV, sASP) summarizing COOP performance (with selected settings) over the collection of human datasets, were computed with the "combined" method [18] and compared with the same statistics calculated for the 14 different programs tested by Tompa and colleagues on human datasets.

## Authors' contributions

SB and GAD conceived the study. SB and AC carried out pattern discovery analyses, developed and tested COOP software and drafted the manuscript. AB and CP participated to the early phases of the work, contributing to gene promoter selection and to the development of the COOP algorithm, respectively. GAD revised the manuscript. All authors read and approved the final manuscript.

## Supplementary Material

Additional File 1 Table 6. Results of the analysis of 26 human positive control datasets with COOP. Click here for file

Additional File 2 Table 7. Comparative evaluation of COOP performance on 26 human positive control datasets. Click here for file

## Figures and Tables

**Table 1:** Description of COOP parameters.

**Step**	**Parameter**	**Description**	**Range**	**Example**
Search for pattern occurrences	*q*	*Quorum *(minimum number out of N input sequences in which a given pattern must be represented)	1 - N	≥ 20 out of 100 sequences

Clustering	*d*	Physical distance between 5'-ends of occurrences of patterns of length p	0 - |p|	≤ 2 nucleotides
	*o*	Ratio between observed overlapping occurrences of two patterns and their average number of occurrences	0 – 1	≥ 0.8

Consensus building	*i*	Ratio between the number of nucleotides per alignment position and the total number of lines in the alignment. The maximal number of adjacent positions exceeding the threshold *i *(*m*) is further analysed for determining the consensus sequence of the motif	0 – 1	≥ 0.5
	*l*	Nucleotide length of the lateral region of the motif	0 - m/2	3 bp (out of 10)
	*f*_*l*_	Frequency of a single nucleotide in each position of the lateral region to be considered specified	0 – 1	≥ 0.6
	*f*_*c*_	Frequency of a single nucleotide in each position of the core region to be considered specified	0 – 1	≥ 0.8

**Table 2:** Procedure for building a consensus sequence starting from a matrix of nucleotide counts, according to selected parameters. Rows from two to five represent the matrix of nucleotide counts in different positions of an alignment associated to a cluster of pattern occurrences. The sixth row contains, for each alignment position, the ratio between number of sequences in the position and the total number of lines in the alignment. Out of 11 positions of the matrix, positions from one to ten (shaded in grey) fulfil the minimum *i *(0.5) and are considered for building the consensus. If the lateral region length is set to 3 nucleotides, a 3-4-3 motif is obtained. The *f*_*l *_(0.6) threshold is applied to the positions in the lateral regions, whereas the *f*_*c *_(0.8) is applied to positions in the core region. Cells containing values fulfilling the condition reported on the left are in bold. In the last row, the derived consensus sequence is shown.

	**1**	**2**	**3**	**4**	**5**	**6**	**7**	**8**	**9**	**10**	**11**
**A**	0	0	0	4	0	0	0	0	0	0	0
**C**	0	0	5	0	5	2	0	0	0	0	2
**G**	0	4	0	0	0	3	5	5	0	4	0
**T**	3	0	0	1	0	0	0	0	5	0	0

*i *(0.5)	**0.6**	**0.8**	**1**	**1**	**1**	**1**	**1**	**1**	**1**	**0.8**	0.4
*f*_*l *_(0.6)	**1**	**1**	**1**					**1**	**1**	**1**	
*f*_*c*_(0.8)				**0.8**	**1**	0.6	**1**				

Consensus sequence	**T**	**G**	**C**	**A**	**C**	**N**	**G**	**G**	**T**	**G**	**-**

**Table 3:** Number of sequences in which most represented patterns were found in different retinal datasets.

**Pattern**	**500U91**	**500PM91**	**500M91**	**1000M91**
**10-2**	44	42	32	40
**12-2**	19	19	10	12
**14-2**	11	11	5	7

**Table 4:** Statistics about patterns found in different groups of retina gene promoter sequences and in the corresponding negative control random datasets.

	***Quorum***	**Pattern**	**Obs.**	**Exp. **	**P-value**
		**10-2**	719	351.3	0.017
	**20**	**12-2**	0	-	-
		**14-2**	0	-	-
**1000M91 **(91 promoters of retinal genes *vs *RAN1000M91_i_)		**10-2**	18683	12846.4	0.016
	**10**	**12-2**	41	35.4	0.324
		**14-2**	0	-	-
	**7**	**12-2**	714	410.2	0.060

**1000M52 **(52 promoters of retinal genes *vs *RAN1000M52_*i*_)	**5**	**12-2**	1537	429.4	0.001

**Table 5:** List of 60 consensus sequences corresponding to selected motifs showing most conserved central regions. For each motif, consensus sequence, length and total number of occurrences in the 1000M dataset are reported, along with LocusLink symbols of corresponding genes. In the last column, for each consensus, the list of mammalian transcription factors recognising similar DNA sequences is reported.

**Consensus sequence**	**Length**	**Total occ.**	**Genes in which promoter the motif was found**	**Transcription factors**
AAAAAAAAAAAAAA	14	151	EFEMP1, CCNI, CNGB3, KCNV2, IMPDH2, SLC24A1, DHRS3, G2AN, RTP801, MGC15WIF11, USH3A, CRX, 18, HMGA1, SLC24A2, RDS, TULP1, DC-TM4F2, OPN1SW, RP1, MGAT4B, GAPD, ELOVL4, RRAD, ARR3	
NGGCCCCGCCCCCN	14	114	EEF1G, HMGA1, EFEMP1, CYBA, KRT18, OPA1, DPYSL4, RAX, FLJ1415, MGC15WIF11, FLJ1415, ALMS1, EIF3S8, G2AN, ALMS1, DC-TM4F2, MSH6, RCV1, KRT19, DHRS3, PITPNC1, RRAD, HPCAL1, MGAT4B, SLC38A3, IMPDH2, CNGB1, RDH5, EFEMP1, CRABP1, C7orf20, CCNI, GNB1, CRX, GAPD, ARF4L, AIPL1, DKFZP564K0822	AP-1, GCF, Sp1, Sp3, TFIID
GCACCCCCAGCCCCN	15	101	RHO, G2AN, EFEMP1, SLCO4A1, CYBA, HPCAL1, KIFC3, RCV1, NK4, KRT18, CRX, ARR3, PPP1R3F, MGAT4B, NRL, RRAD, CCNI, SAG, ALMS1, MGC15WIF11, DKFZP564K0822, VMD2, DPYSL4, GNAT1, GAPD, OPN1SW, RAX, DHRS3, COPEB, SLC38A3, TMEM16B, SLC24A1	Sp1
NGAGGGCAGGGGCNN	15	94	GNB1, KRT19, ELOVL4, VMD2, MSH6, HMGA1, RHO, NK4, SLC38A3, LRRCGUCA1B, CYBA, RCV1, RRAD, GUCY2D, MGC15WIF11, AIPL1, MGAT4B, KIFC3, CRX, CRABP1, G2AN, ALMS1, RTP801, EEF1G, COPEB, OPA1, EFEMP1, KCNV2, PDE6A, AOC2, RLBP1, FLJ1415, RAX, DPYSL4, WIF1, DC-TM4F2	Sp1
CCTCCCTCCCTCCC	14	76	ARF4L, COPEB, RHO, SLC38A3, FLJ1415, WDR17, ELOVL4, DHRS3, KCNV2, OPA1, CCNI, GUCA1B, RDH5, RAX, ALMS1, DKFZP564K0822, NK4, RGS19IP1, RRAD, KIFC3, KRT19, SLCO4A1, HPCAL1, DPYSL4, TNFRSF6, CNGB1, DC-TM4F2	MAZ
NCTCCCCCTCCCCC	14	43	CNGB1, GAPD, RPE65, ALMS1, COPEB, MSH6, RRAD, CRABP1, TNFRSF6, CRX, WIF1, FLJ1415, DKFZP564K0822, PDE6A, RDH5, SLC38A3, CYBA, GNB1, MERTK, WDR17	Sp1, AP-2, MAZ
GNNTGGGGGAGGGGN	15	41	CYBA, RLBP1, KCNV2, CNGB1, COPEB, KIFC3, RDH5, CCNI, FLJ1415, MGC15WIF11, AIPL1, NK4, HPCAL1, CNGB1, GUCA1A, ALMS1	MAZ, Sp1
CNCCCCCACCCCCACC	16	40	RCV1, SLC38A3, HPCAL1, KIFC3, RLBP1, RPE65, DHRS3, RTP801, CYBA, DPYSL4, RDH5, RRAD, COPEB	AP-2alphaB, Sp1, WT1
CTCCCCCTCCCCNNC	15	26	CNGB1, CRX, GAPD, RHO, CNGB1, COPEB, CYBA, AIPL1, RAX	AP-2, MAZ, Sp1
CCCCAGCCCCNCA	13	23	CCNI, EFEMP1, SLCO4A1, MGC15WIF11, ARR3, CYBA, HPCAL1, KIFC3, RAX, RLBP1, MGAT4B, AIPL1, RGS19IP1, ALMS1	Sp1
NNGGCCCCTGCCCN	14	23	HMGA1, NK4, LRRCGUCA1B, FLJ1415, GNB1, KRT19, AIPL1, GUCA1A, DHRS3	Sp1
NCCCCCTCCACCN	13	22	ARR3, HMGA1, KRT19, VMD2, DHRS3, ARF4L, RAX, CCNI, SIRT3, GUCA1B, DC-TM4F2	Sp1
NCNGGGCTGGGGN	13	22	CYBA, HPCAL1, RRAD, GAPD, GUCA1A, RHO, G2AN, EFEMP1	Sp1
NNTCCCCCTCCCNN	14	22	TNFRSF6, CNGB1, CRX, EEF1G, GAPD, RPE65, ALMS1, DKFZP564K0822, COPEB, AIPL1	AP-2alphaB, MAZ, Sp1, WT1 -KTS
NNCCCAGCCCCCAN	14	20	RDH5, SLC38A3, EFEMP1, ARR3, CYBA, GAPD, HPCAL1, NK4, PPP1R3F	Sp1
NTGGGGGAGGGGNA	14	20	COPEB, CYBA, RLBP1, PITPNC1, CNGB1, CRX, GAPD, MERTK, CCNI	MAZ, Sp1, Sp3
CCNGCCCTGGCCT	13	18	GUCA1A, GUCY2D, RCV1, VMD2, EFEMP1, LRRCGUCA1B, C7orf20, 4, RRAD, UNC119, MERTK	Sp1
GCNGCCCCTGCCN	13	18	CRX, CYBA, GNB1, HMGA1, RHO, SLC38A3, MGAT4B, FLJ1415, KRT18	
NCNGGGGGCGGGG	13	18	CYBA, RRAD, FLJ1415, HMGA1, RDH5, RGS19IP1, G2AN, RTP801, DC-TM4F2	AP-1, ER, Sp1
CTNCCCCTCCCC	12	17	RLBP1, AIPL1, PITPNC1, CNGB1, GAPD, RHO, CNGB1, EFEMP1, COPEB, CYBA, GNB1, PDE6A	AP-2alphaB, MAZ, Sp1
GGGGTGGGGNTG	12	17	GUCY2D, FLJ1415, AIPL1, RDH5, CRABP1, HPCAL1, KIFC3, DHRS3, RTP801, CYBA, RLBP1	AP-2alphaB, Sp1, Sp3
CCCGCCCCTGNCC	13	16	GNB1, HPCAL1, KRT19, MGAT4B, G2AN,	Sp1
NGGGGGTGGGGGN	13	16	HPCAL1, RRAD, DHRS3, FLJ1415, CYBA, GNB1, DPYSL4	Sp1
NNCCCCCGCCCCNN	14	16	GNB1, RGS19IP1, LRRCGUCA1B, ALMS1, DC-TM4F2, KRT18, SAG	AP-1, AP-2alphaB, ER, Krox-20, Sp1, WT1, WT1 I, WT1 I -KTS
AGNGGGAGGGGCN	13	14	CYBA, EFEMP1, RAX, MGC15WIF11, ARF4L, CRX, SLCO4A1	MAZ, Sp1, Sp3
CCCTGTCCCTGGAN	14	14	ARR3, HPCAL1, FLJ1415, DC-TM4F2, KRT19, LRRCGUCA1B, TMEM16B	GR
CGGGGCCGCCNCN	13	14	FLJ1415, DC-TM4F2, MGC15WIF11, COPEB, MGAT4B, SLCO4A1, RAX	CUP, Sp1
CTCTCTCTCCNTN	13	14	GAPD, GUCA1A, NRL, RRAD, FLJ1415, GNAT2, KCNV2	
NANCTCTGCACCC	13	14	LRAT, TNFRSF6, CYBA, KIFC3, DPYSL4, G2AN, RTP801	
NCCGCCCCCGCCN	13	14	GNB1, IMPDH2, SLC38A3, COPEB, CYBA, KRT18, SLCO4A1	AP-1, ER, Kxox-20, Sp1, WT1 I -KTS, WT1-del2
NGGCCTCTGGNCN	13	14	CYBA, GAPD, KRT19, RDH5, DPYSL4, HPCAL1, MGAT4B	
NGGGAGGGGGAAG	13	14	GAPD, AIPL1, FLJ1415, EEF1G, RPE65, ALMS1, WDR17	AP-2alphaB, MAZ, Sp1, WT1 I -KTS
NGNCCCCAGCCCC	13	14	GAPD, GUCA1A, RHO, ARR3, CYBA, NK4, PPP1R3F	AP-2, Sp1
NNCCCAGCCCAGNN	14	14	GAPD, RHO, ARR3, CRABP1, CYBA, RRAD, MGAT4B	Sp1
TGGGGGTGGGGGN	13	14	HPCAL1, RLBP1, DHRS3, CYBA, HMGA1, RRAD, DPYSL4	Sp1
NGGCGGGGGCGGGG	14	13	EFEMP1, KRT18, RRAD, SLCO4A1, IMPDH2, EFEMP1, COPEB	AP-1, Krox-20, Sp1, WT1 I -KTS, WT1-del2
GGNAGGGGCGGG	12	11	ELOVL4, REA, G2AN, GNB1, MSH6, GUCY2D, RGS19IP1, LRRC21, SLCO4A1, PITPNC1	MAZ, Sp1
CCCGCCCGCCCC	12	9	GNB1, RGS19IP1, WIF1, PITPNC1, DC-TM4F2, HMGA1, DPYSL4, KRT18, RAX	Sp1
GGGCGGGGCNGG	12	9	CYBA, DPYSL4, MGAT4B, MSH6, RCV1, ALMS1, FLJ1415	ER, GCF, Sp1
GGGCTGGGGGTG	12	9	CYBA, HPCAL1, KIFC3, RCV1, RHO, G2AN, DKFZP564K0822	Sp1
GGGGAAGGGNGG	12	9	TULP1, CRX, MSH6, KRT19, CNGB1, SLC38A3, AIPL1, HMGA1, FLJ1415	
GGGGCGGGCNNG	12	9	EEF1G, KRT19, DC-TM4F2, GUCY2D, RGS19IP1, PITPNC1, C7orf20, RTP801	ER, Sp1
GGNGCGGGCGGG	12	9	HMGA1, KRT19, DPYSL4, DC-TM4F2, RGS19IP1, WIF1, PITPNC1, FLJ1415	AP-2, ETF, Krox-20, Sp1, WT1 I -KTS
GNNGGGGCTGGG	12	9	GAPD, HPCAL1, KIFC3, RCV1, RAX, COPEB, RDH5	WT1 -KTS
CAGGGGGCGGGG	12	8	CYBA, EFEMP1, HPCAL1, FLJ1415, GAPD, HMGA1, G2AN, DC-TM4F2	AP-1, ER, Sp1, Yi
CNCCCCCACCCC	12	8	CYBA, HMGA1, RCV1, SLC38A3, HPCAL1, RLBP1, DHRS3	AP-2alphaB, CACCC-binding, factor, Sp1, WT1
GAGTGGGGGAGG	12	8	DHRS3, KCNV2, COPEB, CYBA, HMGA1, WIF1, FLJ1415, MGC15WIF11	
GCCTGGGGGAGG	12	8	CYBA, SIRT3, KIFC3, CCNI, DKFZP564K0822, DC-TM4F2, MGC15WIF11	AP-2
GGGCAGGGGCNG	12	8	CYBA, GNB1, HPCAL1, HMGA1, RHO, SLC38A3, MGAT4B, G2AN	Sp1
GGGCGGGGCTGG	12	8	CYBA, HPCAL1, RAX, MSH6, RCV1, ALMS1, DC-TM4F2	ER, GCF, Sp1
CCCTGTCCCTGG	12	7	CNGB1, GNB1, FLJ1415, KRT19, ELOVL4, TMEM16B, FLJ1415	GR
CCTTCCCCCNGC	12	7	GNB1, SLC38A3, AIPL1, SLCO4A1, RDH5, TULP1, NK4	MAZ
CNCCTCCTGCNC	12	7	CRABP1, GUCA1A, PDE6A, RGR, DPYSL4, WIF1, HPCAL1	PPUR, Sp1
CNGCCCCCAGNC	12	7	RHO, EFEMP1, DC-TM4F2, CNGB1, CYBA, NK4, MERTK	Sp1
GCNCCCCTCCCC	12	7	COPEB, CRX, HPCAL1, RGR, CNGB1, MERTK, RAX	MAZ, Sp1
GGGCAGGGGCGG	12	7	ELOVL4, HMGA1, HPCAL1, RHO, SLC38A3, MGAT4B, G2AN	Sp1
GGGGCTGGGGNC	12	7	ARR3, CYBA, HPCAL1, NK4, RAX, PPP1R3F, RLBP1	AP-2alphaB, Sp1
GNAGGGGGCAGG	12	7	GAPD, NK4, GUCA1B, SLC38A3, WIF1, G2AN, EFEMP1	Sp1
TGGGGGAGGNNA	12	7	KCNV2, COPEB, HMGA1, KIFC3, RDH5, CCNI, FLJ1415	MAZ, Sp1
TTTTTTTTTNTA	12	7	IMPDH2, G2AN, SLC24A2, RTP801, KCNV2, USH3A-PROMB, CCNI	TBP
