# Supplementary material for: A multistep bioinformatic approach detects putative regulatory elements in gene promoters
Source: BMC Bioinformatics. 2005 May 18;6:121. doi: 10.1186/1471-2105-6-121 (PMC1173081; doi:10.1186/1471-2105-6-121)
Supplement: Additional File 1 — Table 6. Results of the analysis of 26 human positive control datasets with COOP. [file 1471-2105-6-121-S1.doc]

**Table 6. Results of the analysis of 26 human positive control datasets with COOP.**

For each dataset (first column), among predicted motifs, only the one represented in the highest number of sequences was selected. In columns from 2 to 6 are reported the length and the number of sequences in the sample, the number of known signals and the number of known signals shorter than 12 bp (asterisks indicate that more than one half of known signals were shorter than 12 bp). For each dataset, the overlap between pattern occurrences belonging to the cluster, corresponding to the selected motif, and known signals was evaluated both at nucleotide-level and at site-level (columns from 7 to 13). We used the measures of accuracy proposed by Tompa and colleagues [18] (see Methods), which are reported in the last 8 columns.

| Dataset | sequence length | # sequences | quorum | # signals | # signals < 12 bp | nTP | nFP | nFN | nTN | sTP | sFP | sFN | nSn | nPPV | nSp | nPC | nCC | sSn | sPPV | sASP |
| --- | --- | --- | --- | --- | --- | --- | --- | --- | --- | --- | --- | --- | --- | --- | --- | --- | --- | --- | --- | --- |
| **hm01g** | 2000 | 18 | 5 | 16 | *11 | 0 | 95 | 236 | 35669 | 0 | 7 | 16 | 0 | 0 | 0.9973 | 0 | -0.0042 | 0 | 0 | 0 |
| **hm02r** | 1000 | 9 | 3 | 11 | 3 | 13 | 73 | 244 | 8670 | 1 | 5 | 10 | 0.0506 | 0.1512 | 0.9917 | 0.0394 | 0.0723 | 0.0909 | 0.1667 | 0.1288 |
| **hm03r** | 1500 | 10 | 3 | 15 | 0 | 12 | 230 | 396 | 14362 | 1 | 13 | 14 | 0.0294 | 0.0496 | 0.9842 | 0.0188 | 0.0176 | 0.0667 | 0.0714 | 0.069 |
| **hm04m** | 2000 | 13 | 3 | 11 | *7 | 0 | 174 | 168 | 25658 | 0 | 13 | 11 | 0 | 0 | 0.9933 | 0 | -0.0066 | 0 | 0 | 0 |
| **hm05r** | 1000 | 3 | 2 | 11 | 3 | 15 | 145 | 178 | 2662 | 1 | 10 | 10 | 0.0777 | 0.0938 | 0.9483 | 0.0444 | 0.0285 | 0.0909 | 0.0909 | 0.0909 |
| **hm06g** | 500 | 9 | 3 | 9 | *6 | 0 | 42 | 75 | 4383 | 0 | 3 | 9 | 0 | 0 | 0.9905 | 0 | -0.0126 | 0 | 0 | 0 |
| **hm07m** | 1000 | 5 | 2 | 6 | 2 | 0 | 62 | 127 | 4811 | 0 | 4 | 6 | 0 | 0 | 0.9873 | 0 | -0.0181 | 0 | 0 | 0 |
| **hm08m** | 500 | 15 | 3 | 13 | 6 | 4 | 106 | 194 | 7196 | 1 | 8 | 12 | 0.0202 | 0.0364 | 0.9855 | 0.0132 | 0.0076 | 0.0769 | 0.1111 | 0.094 |
| **hm09g** | 1500 | 10 | 3 | 10 | 3 | 0 | 95 | 160 | 14745 | 0 | 6 | 10 | 0 | 0 | 0.9936 | 0 | -0.0083 | 0 | 0 | 0 |
| **hm10m** | 500 | 6 | 2 | 11 | *11 | 0 | 56 | 89 | 2855 | 0 | 3 | 11 | 0 | 0 | 0.9808 | 0 | -0.0241 | 0 | 0 | 0 |
| **hm11g** | 1000 | 8 | 2 | 19 | *11 | 24 | 968 | 245 | 6763 | 3 | 60 | 17 | 0.0892 | 0.0242 | 0.8748 | 0.0194 | -0.0197 | 0.15 | 0.0476 | 0.0988 |
| **hm12r** | 500 | 2 | 2 | 5 | 2 | 0 | 40 | 70 | 890 | 0 | 3 | 5 | 0 | 0 | 0.957 | 0 | -0.056 | 0 | 0 | 0 |
| **hm13r** | 1000 | 6 | 2 | 9 | 0 | 0 | 153 | 164 | 5683 | 0 | 11 | 9 | 0 | 0 | 0.9738 | 0 | -0.0271 | 0 | 0 | 0 |
| **hm14r** | 1000 | 2 | - | 4 | 0 | 0 | 0 | 82 | 5918 | 0 | 0 | 4 | 0 | NaN | 1 | 0 | NaN | 0 | NaN | NaN |
| **hm15r** | 2000 | 4 | 2 | 4 | 0 | 0 | 132 | 257 | 7611 | 0 | 9 | 4 | 0 | 0 | 0.983 | 0 | -0.0236 | 0 | 0 | 0 |
| **hm16g** | 3000 | 7 | 7 | 7 | 1 | 0 | 168 | 164 | 20668 | 0 | 12 | 7 | 0 | 0 | 0.9919 | 0 | -0.008 | 0 | 0 | 0 |
| **hm17g** | 500 | 11 | 3 | 10 | 1 | 114 | 12 | 30 | 5344 | 8 | 1 | 2 | 0.7917 | 0.9048 | 0.9978 | 0.7308 | 0.8425 | 0.8 | 0.8889 | 0.8444 |
| **hm18m** | 3000 | 5 | 2 | 7 | *4 | 0 | 407 | 96 | 14497 | 0 | 27 | 7 | 0 | 0 | 0.9727 | 0 | -0.0134 | 0 | 0 | 0 |
| **hm19g** | 500 | 5 | 2 | 4 | 0 | 29 | 156 | 58 | 2257 | 2 | 11 | 2 | 0.3333 | 0.1568 | 0.9354 | 0.1193 | 0.1881 | 0.5 | 0.1538 | 0.3269 |
| **hm20r** | 2000 | 35 | 8 | 76 | 29 | 135 | 301 | 1181 | 68383 | 11 | 22 | 65 | 0.1026 | 0.3096 | 0.9956 | 0.0835 | 0.1695 | 0.1447 | 0.3333 | 0.239 |
| **hm21g** | 1000 | 5 | 2 | 7 | *5 | 1 | 167 | 92 | 4740 | 0 | 10 | 7 | 0.0108 | 0.006 | 0.966 | 0.0038 | -0.0175 | 0 | 0 | 0 |
| **hm22m** | 500 | 6 | 2 | 5 | 1 | 30 | 14 | 76 | 2880 | 2 | 1 | 3 | 0.283 | 0.6818 | 0.9952 | 0.25 | 0.4272 | 0.4 | 0.6667 | 0.5333 |
| **hm23r** | 500 | 4 | 2 | 5 | 0 | 0 | 143 | 46 | 1811 | 0 | 3 | 5 | 0 | 0 | 0.9268 | 0 | -0.0426 | 0 | 0 | 0 |
| **hm24m** | 500 | 8 | 2 | 8 | 4 | 11 | 36 | 81 | 3872 | 1 | 2 | 7 | 0.1196 | 0.234 | 0.9908 | 0.0859 | 0.1535 | 0.125 | 0.3333 | 0.2292 |
| **hm25g** | 500 | 2 | - | 5 | 2 | 0 | 0 | 70 | 930 | 0 | 0 | 5 | 0 | NaN | 1 | 0 | NaN | 0 | NaN | NaN |
| **hm26m** | 1000 | 9 | 3 | 10 | 2 | 7 | 65 | 240 | 8688 | 1 | 4 | 9 | 0.0283 | 0.0972 | 0.9926 | 0.0224 | 0.0384 | 0.1 | 0.2 | 0.15 |
| **Human** |  |  |  |  |  | **395** | **3840** | **4819** | **281946** | **32** | **248** | **267** | **0.0758** | **0.0933** | **0.9866** | **0.0436** | **0.069** | **0.107** | **0.1143** | **0.1107** |
