# Supplementary material for: A multistep bioinformatic approach detects putative regulatory elements in gene promoters
Source: BMC Bioinformatics. 2005 May 18;6:121. doi: 10.1186/1471-2105-6-121 (PMC1173081; doi:10.1186/1471-2105-6-121)
Supplement: Additional File 2 — Table 7. Comparative evaluation of COOP performance on 26 human positive control datasets. [file 1471-2105-6-121-S2.doc]

**Table 7. Comparative evaluation of COOP performance on 26 human positive control datasets.**

Measures of accuracy of COOP and of 14 different tools previously included in the evaluation by Tompa and colleagues [18] over the collection of 26 human data sets were summarized by the combined method. For each tool, the sums of nTP, nFP, nFN, nTN, sTP, sFP and sFN over the data were calculated and the statistical measures (nSn, nPPV, nSp, nPC, nCC, sSn, sPPV, sASP) were computed as though the collection of human datasets was one large dataset. Since this method tends to reward programs that make no predictions on many datasets, the second column shows the number of human datasets, out of 26, for which no motif was predicted by each given tool.

| Tool | Non predicted | nSn | nPPV | nSp | nPC | nCC | sSn | sPPV | sASP |
| --- | --- | --- | --- | --- | --- | --- | --- | --- | --- |
| **COOP** | 2 | 0,0758 | 0,0933 | 0,9866 | 0,0436 | 0,0690 | 0,1070 | 0,1143 | 0,1107 |
| **AlignACE** | 17 | 0,0393 | 0,1026 | 0,9938 | 0,0292 | 0,0531 | 0,0738 | 0,1236 | 0,0987 |
| **ANN-Spec** | 1 | 0,0903 | 0,1032 | 0,9859 | 0,0506 | 0,0813 | 0,1644 | 0,0984 | 0,1314 |
| **Consensus** | 26 | 0 | NaN | 1,0000 | 0,0000 | NaN | 0,0000 | NaN | NaN |
| **GLAM** | 2 | 0,0236 | 0,0368 | 0,9888 | 0,0146 | 0,0155 | 0,0403 | 0,0600 | 0,0501 |
| **Improbizer** | 0 | 0,0416 | 0,0476 | 0,9850 | 0,0227 | 0,0284 | 0,0705 | 0,0484 | 0,0594 |
| **MEME** | 2 | 0,0381 | 0,0604 | 0,9893 | 0,0239 | 0,0344 | 0,0604 | 0,0811 | 0,0707 |
| **MEME3** | 8 | 0,0420 | 0,0471 | 0,9847 | 0,0227 | 0,0282 | 0,0638 | 0,0788 | 0,0713 |
| **MITRA** | 0 | 0,0244 | 0,0471 | 0,9911 | 0,0163 | 0,0215 | 0,0403 | 0,0469 | 0,0436 |
| **MotifSampler** | 0 | 0,0250 | 0,0417 | 0,9896 | 0,0159 | 0,0188 | 0,0470 | 0,0431 | 0,0450 |
| **oligo/dyad-analysis** | 0 | 0,0371 | 0,2140 | 0,9975 | 0,0327 | 0,0826 | 0,0604 | 0,1500 | 0,1052 |
| **QickScore** | 14 | 0,0051 | 0,0099 | 0,9909 | 0,0034 | -0,0056 | 0,0000 | 0 | 0 |
| **SeSiMCM** | 0 | 0,0459 | 0,0280 | 0,9713 | 0,0177 | 0,0135 | 0,0671 | 0,0631 | 0,0651 |
| **Weeder** | 10 | 0,0543 | 0,2747 | 0,9974 | 0,0475 | 0,1155 | 0,1074 | 0,2581 | 0,1827 |
| **YMF** | 4 | 0,0410 | 0,0967 | 0,9931 | 0,0297 | 0,0521 | 0,0738 | 0,0803 | 0,0771 |
